# Magnetic-Stimulation-Related Physiological Artifacts in Hemodynamic Near-Infrared Spectroscopy Signals

**DOI:** 10.1371/journal.pone.0024002

**Published:** 2011-08-26

**Authors:** Tiina Näsi, Hanna Mäki, Kalle Kotilahti, Ilkka Nissilä, Petri Haapalahti, Risto J. Ilmoniemi

**Affiliations:** 1 Department of Biomedical Engineering and Computational Science (BECS), Aalto University School of Science, Espoo, Finland; 2 BioMag Laboratory, HUSLAB, Helsinki University Central Hospital, Helsinki, Finland; 3 Division of Clinical Physiology and Nuclear Medicine, HUSLAB, Helsinki University Central Hospital, Helsinki, Finland; Cuban Neuroscience Center, Cuba

## Abstract

Hemodynamic responses evoked by transcranial magnetic stimulation (TMS) can be measured with near-infrared spectroscopy (NIRS). This study demonstrates that cerebral neuronal activity is not their sole contributor. We compared bilateral NIRS responses following brain stimulation to those from the shoulders evoked by shoulder stimulation and contrasted them with changes in circulatory parameters. The left primary motor cortex of ten subjects was stimulated with 8-s repetitive TMS trains at 0.5, 1, and 2 Hz at an intensity of 75% of the resting motor threshold. Hemoglobin concentration changes were measured with NIRS on the stimulated and contralateral hemispheres. The photoplethysmograph (PPG) amplitude and heart rate were recorded as well. The left shoulder of ten other subjects was stimulated with the same protocol while the hemoglobin concentration changes in both shoulders were measured. In addition to PPG amplitude and heart rate, the pulse transit time was recorded. The brain stimulation reduced the total hemoglobin concentration (HbT) on the stimulated and contralateral hemispheres. The shoulder stimulation reduced HbT on the stimulated shoulder but increased it contralaterally. The waveforms of the HbT responses on the stimulated hemisphere and shoulder correlated strongly with each other (*r* = 0.65–0.87). All circulatory parameters were also affected. The results suggest that the TMS-evoked NIRS signal includes components that do not result directly from cerebral neuronal activity. These components arise from local effects of TMS on the vasculature. Also global circulatory effects due to arousal may affect the responses. Thus, studies involving TMS-evoked NIRS responses should be carefully controlled for physiological artifacts and effective artifact removal methods are needed to draw inferences about TMS-evoked brain activity.

## Introduction

Transcranial magnetic stimulation (TMS) activates the brain in a direct and controlled manner [1]; the location, timing, amplitude, direction, and wave shape of the TMS-induced current in the brain can be accurately determined. The TMS-evoked neuronal activity is coupled to brain hemodynamics through neurovascular coupling. Increased neuronal activity leads to increased blood flow, oxygenation and volume in the affected regions. This *hemodynamic response* can be recorded with near-infrared spectroscopy (NIRS) [2]–[10], functional magnetic resonance imaging (fMRI) [11], [12], or positron emission tomography (PET) [13]–[15]. The TMS-evoked hemodynamic responses inform us about the neurovascular coupling, neuronal plasticity, functional connectivity between brain regions, and the effects of TMS in the treatment of neurological and psychiatric diseases [16], [17]. TMS–NIRS has several advantages: NIRS is not disturbed electromagnetically by TMS, the temporal resolution is better than in PET and fMRI, allowing the shape of the hemodynamic response to be obtained, and the subjects are not exposed to ionizing radiation.

TMS-evoked NIRS responses have been reported previously, but the question to what extent they reflect TMS-evoked cerebral hemodynamic responses has not been critically addressed. TMS induces currents also in other excitable cells than just cerebral neurons and can activate them (see the physical principles of TMS in, e.g., [18]). The activation of muscles or sympathetic neurons can produce local changes in blood flow, volume and oxygenation. These types of temporally and spatially confined hemodynamic changes that are not caused by TMS-evoked cerebral activity may occur in both the brain and the extracerebral layers. In addition to local effects of TMS, stimulation-related changes in systemic circulation may arise [19]–[21], for instance, due to discomfort and changes in arousal state. Since the NIRS measurement is sensitive to hemodynamic changes also in extracerebral tissue, it is affected by systemic circulation. Both systemic changes and local direct effects of TMS on circulation may produce physiological artifacts, which mask the cerebral hemodynamic response [22]–[24].

Sham stimulation is often implemented by moving the TMS coil away from the head, which decreases the strength of the magnetic field and currents induced in the tissue. However, the traditional sham stimulation is not a suitable control for stimulation-related effects in NIRS since the local tissue effects, the discomfort and the cerebral effect all depend on the TMS-induced currents in the tissue [18]. In this study, we characterize stimulation-related physiological artifacts in NIRS signals by comparing TMS-evoked bilateral NIRS responses measured during and after primary motor cortex (M1) stimulation with those evoked by shoulder stimulation and measured in the shoulders. In addition, we contrast the NIRS responses with changes in circulatory parameters.

## Methods

### Ethics Statement

All participants gave their written informed consent before the experiment. The study was accepted by the Ethics Committee of Helsinki University Central Hospital and was in compliance with the declaration of Helsinki.

### Participants

Thirteen healthy subjects (age 22–32, mean 27; 1 female, 2 left-handed) participated in brain stimulation experiments (“brain subjects”) and ten different healthy subjects (22–33, mean 26; 2 female) in shoulder stimulation experiments (“shoulder subjects”). None of the subjects had any history of neurological or cardiac diseases nor were they taking any medication affecting their nervous system. Two male brain subjects were excluded because of excessive movement and one because of difficulty staying awake.

The brain subjects sat on a reclining chair in a dimmed room in a half-sitting position and the shoulder subjects in an upright position to assure the best position for giving TMS. They were instructed to stay relaxed and to keep their eyes open. To prevent an auditory response, the brain subjects listened to masking white noise (volume below 90 dB) through noise-damping headphones adjusted so that they did not perceive the coil click. The shoulder subjects watched a silent movie during the stimulation and wore hearing protection. Masking noise was not considered necessary because the auditory responses from the brain could not directly affect the NIRS signals of the shoulder subjects. Both subject groups could sense the stimulation because of skin sensory fiber stimulation.

### Magnetic Stimulation

**Figure 1:**
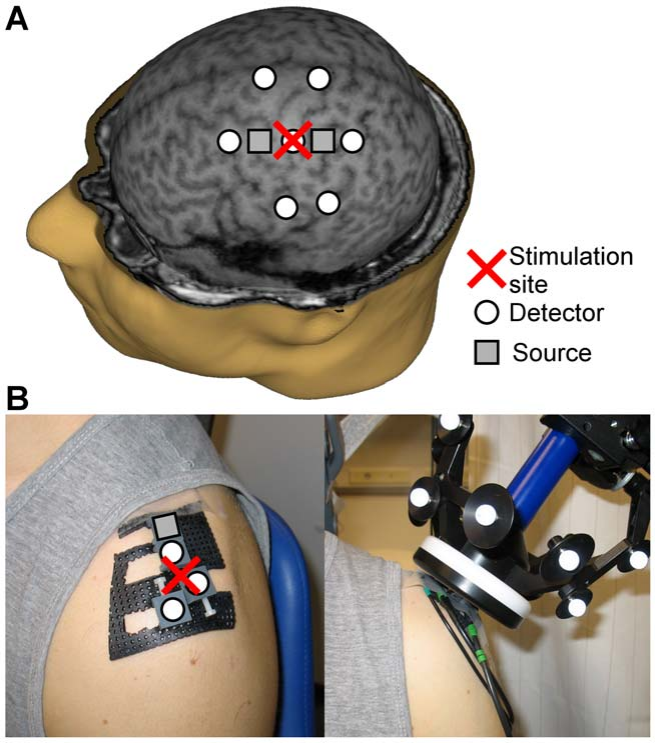
Measurement setup. The position of the NIRS probe (A) in the brain experiments digitized with the NBS software and (B) in the shoulder experiments without (left) and with (right) the stimulation coil. Three different source-to-detector distances (1.3, 2.8, and 3.8 cm) were used to estimate signals originating in different proportions from different depths of the measured tissue. Identical probes were attached on the contralateral hemisphere and shoulder.

An eXimia stimulator with its figure-of-8 biphasic coil (average winding diameter 50 mm; Nexstim Ltd., Helsinki, Finland) was used to stimulate the left M1 hand area of the brain subjects (Figure 1A) with eight-second repetitive TMS (rTMS) trains at 0.5, 1, and 2 Hz. 25 trains at each frequency were given in randomized order, interleaved with 28–38-s rest periods. The coil location and orientation were determined with the MRI-guided Nexstim eXimia Navigated Brain Stimulation system (NBS) and adjusted further to produce maximal responses from the abductor pollicis brevis (APB). The stimulation intensity was 75% of the resting motor threshold of the APB, which was assessed by recording motor-evoked potentials with the ME6000 EMG device and MegaWin software (Mega Electronics Ltd., Kuopio, Finland). A subthreshold intensity was selected in order to avoid recording somatosensory responses to TMS-evoked thumb movement with NIRS. Even below the motor threshold, TMS is known to elicit cerebral neuronal [25] and hemodynamic activity [13], [15], [26]–[28] and NIRS responses [2]–[4], [7].

In the shoulder experiments, magnetic pulse trains identical to those in the brain experiments were delivered above the proximal end of the left humerus (Figure 1B). This stimulation site was chosen because there is bone and no muscular tissue under the site. A nearby muscle (deltoid) may be activated to some extent like the temporal muscle may be activated during the stimulation of M1. Since the brain subjects did not report cranial muscle activation, and as the goal was to produce similar effects in both shoulder and brain stimulation, the position of the coil was adjusted slightly if the shoulder subjects reported muscle contractions. The maximal induced current was directed medially and the intensity was 57% of the maximal stimulator output (equal to the average intensity for the brain subjects), corresponding to 969 V charge of the capacitor and 3.1 kA current in the stimulator coil.

### NIRS Recordings

A frequency-domain instrument with two time-multiplexed laser diodes modulated at 100 MHz recorded the NIRS signals [29]. The optical power was 4–12 mW at the surface of the tissue. One NIRS probe was attached over each hemisphere or shoulder. Each probe comprised two source fibers and seven detector fiber bundles (brain experiments, Figure 1A) or one source and three detectors (shoulder experiments, Figure 1B). The fibers had three different source-to-detector distances in both probe types: short (1.3 cm), intermediate (2.8 cm), and long (3.8 cm). The purpose of the different source-to-detector distances was to provide signals with different relative contributions from superficial and brain tissues. Signals measured at the shortest source-to-detector distance have a negligible relative contribution from the brain [30]. The source fibers and detector fiber bundles had prism terminals to minimize the thickness of the probes (approximately 1 cm), which enabled coil positioning close to the stimulated tissue. The head probes were positioned with the NBS system so that the central detectors were located above the hand areas of the M1 on both hemispheres (Figure 1A). The shoulder probes were placed so that the stimulation site was between the short- and intermediate-distance detectors, the source being medial and the long-distance detector lateral to it (Figure 1B). Because of the differences in head and shoulder anatomy, only reproducing the source-to-detector distances but not the overall layout of the fibers between the brain and shoulder experiments was considered meaningful.

### Recordings of systemic data

Photoplethysmographic (PPG) pulse waveforms were recorded with a pulse oximeter (S/5 patient monitor, Datex-Ohmeda, Finland) attached to the left index finger of both brain and shoulder subjects. In the shoulder subjects, the S/5 monitor simultaneously recorded an electrocardiogram (ECG). All subjects had a movement sensor (inclinometer) attached to their head (brain subjects) or the right shoulder (shoulder subjects).

### Data Analysis

To attenuate drifts and artifacts due to fiber contact variations, the NIRS amplitude signals were detrended by dividing them with a lowpass-filtered version of the corresponding signal (−3-dB cutoff at 0.015 Hz). High-frequency noise was suppressed by lowpass filtering (−3-dB cutoff at 0.5 Hz). The amplitude signals were converted into total hemoglobin (HbT) and oxy- and deoxyhemoglobin (HbO_2_ and HbR, presented as supporting information) concentrations with the modified Beer–Lambert law and a differential pathlength factor of 6 [31]. The sampling frequency of the concentrations was 2 Hz. Epochs containing peak-to-peak changes greater than six times the standard deviation of the channel were rejected as they most likely contained motion artifacts or changes in the contact between the probe and the skin. Also epochs with movements as shown by the inclinometer signal were rejected.

The heart rate and the PPG peak-to-peak amplitude were determined from the PPG for the brain and the shoulder subjects. In addition, for the shoulder subjects, the pulse transit time (PTT) was determined from the ECG and the PPG. The PPG amplitude reflects the amount of blood pulsating in the blood vessels of the finger. It depends on the local vascular compliance and is affected by vasoconstriction and -dilation [32]. The PTT was defined as the time difference between the R peak in the ECG and the corresponding PPG pulse wave peak. It represents the time taken by the pulse pressure wave to travel from the heart to the finger and thus characterizes arterial stiffness along the path that the pressure wave travels. The PTT and the PPG amplitude depend on both systemic and local vascular tone and closely follow circulatory changes. The inverse of the pulse transit time (1/PTT), which correlates with blood pressure [33], was analyzed subsequently. The heart rate, PPG amplitude and 1/PPT signals were interpolated to a sampling rate of 2 Hz. In addition, the PPG amplitudes were divided by the mean value of each subject as they depend on the size of the blood vessels in the sampling volume and thus vary between subjects. Epochs rejected from the NIRS signals were also rejected from the heart rate, PPG amplitude, and 1/PTT signals. Epochs having peak-to-peak changes eight times the standard deviation of the averaged response were also rejected (at most 3 epochs per subject).

The HbT signals, as well as heart rate, PPG amplitude, and 1/PTT were averaged over baseline-corrected epochs ranging from −2 to 25 s with respect to the onset of the pulse train. The averages for each stimulation frequency were calculated over the subjects and, in the brain experiments, over channels with identical source-to-detector distances within each hemisphere.

### Statistical Methods

To test if the responses differed significantly from baseline, paired *t*-tests were applied to compare the amplitudes averaged over the 2-s time interval at the end of the magnetic pulse train (6…8 s after the stimulation onset) with the average amplitudes of the baseline (−2…0 s). To correct for multiple comparisons, the significance level *α* = 0.05 was adjusted for positively correlated tests by controlling the false discovery rate (FDR) [34]. The number of tests for correcting the significance level was 36 for HbT, HbO_2_ and HbR (3 frequencies×3 source-to-detector distances×2 hemispheres/sides×2 stimulation sites, i.e., brain and shoulder), 6 for the heart rate and PPG amplitude (3 frequencies×2 stimulation sites), and 3 for the 1/PTT (3 frequencies).

To compare the HbT waveforms from the brain with those from the shoulder and with the circulatory responses, Pearson's correlation coefficients (*r*) were calculated between the corresponding values in the time period from 0 to 25 s after stimulation onset. In each comparison, the stimulation frequencies and the source-to-detector distances were matched. The HbT signals from the shoulder were similarly compared with the circulatory responses.

## Results

**Figure 2:**
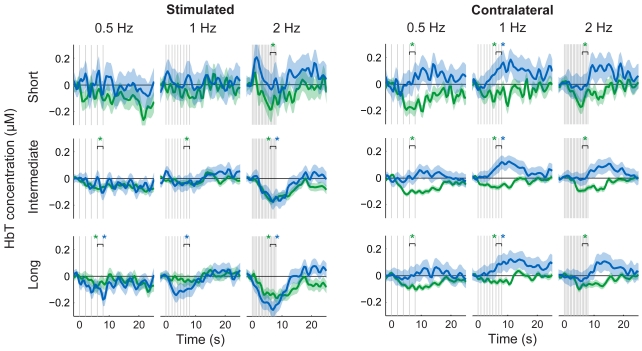
HbT responses following brain (green) and shoulder (blue) stimulation. HbT responses from the stimulated (left) and the contralateral (right) brain hemispheres and shoulders at short (uppermost row), intermediate (center row), and long (lowest row) source-to-detector distance channels. The standard errors of mean are shaded with the corresponding color. Vertical lines indicate times at which the magnetic pulses were given. HbT decreased on both the stimulated brain hemisphere and shoulder, while the brain and shoulder responses had opposite polarities on the contralateral side. * *p*<0.05 (*t*-tests for the response amplitudes compared to baseline, *p*-values controlled for FDR).

On the stimulated side, in both brain and shoulder experiments, the 2-Hz stimulation decreased the HbT concentration significantly in channels with intermediate and long source-to-detector distances (Figure 2); the waveforms in the brain and the shoulder correlated strongly with each other (at intermediate distance: *r* = 0.87; at long distance: *r* = 0.65). Contralaterally, HbT concentrations decreased in response to brain stimulation but increased in response to shoulder stimulation (Figure 2). The changes in HbT resulted mostly from changes in HbO_2_ in the brain subjects, whereas HbO_2_ and HbR changed by approximately the same amount in the shoulder subjects (Figures S1 and S2).

**Figure 3:**
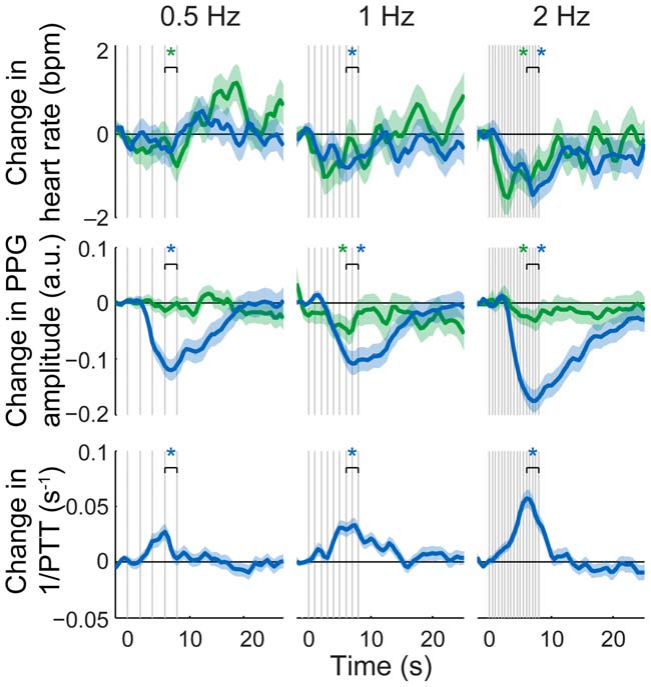
Changes in circulatory parameters following brain (green) and shoulder (blue) stimulation. The standard errors of mean are shaded with the corresponding color. Vertical lines indicate times at which the magnetic pulses were given. The PPG amplitude and heart rate decreased and 1/PTT increased in response to stimulation. * *p*<0.05 (*t*-tests for the response amplitudes compared to baseline, *p*-values controlled for FDR).

All the circulatory parameters were affected by the stimulation (Figure 3); in general, the heart rate and PPG amplitude (reflecting local vascular compliance) decreased while 1/PTT (reflecting blood pressure) increased. The HbT concentrations showed intermediate to strong correlations with the PPG amplitude in cases where both responses compared were statistically significant (range of correlation coefficients on the stimulated hemisphere: *r* = 0.34…0.46; on the contralateral hemisphere: *r* = 0.26…0.65; on the stimulated shoulder: *r* = 0.49…0.83; on the contralateral shoulder: *r* = −0.86…−0.53). In these cases, many of the HbT responses showed intermediate to strong correlation also with the heart rate (on the contralateral hemisphere: *r* = 0.31…0.50; on the stimulated shoulder: *r* = 0.49…0.65) and the 1/PTT waveforms (on the stimulated shoulder: *r* = −0.89…−0.32), while the correlation coefficients between other responses varied greatly between channels and conditions (with heart rate and on the stimulated hemisphere: *r* = −0.13…0.49; with 1/PTT and on the contralateral shoulder: *r* = 0.01…0.47) or did not show a notable correlation (with heart rate and on the contralateral shoulder: *r* = −0.09…0.11).

## Discussion

We recorded magnetically evoked hemoglobin concentration decreases in the stimulated shoulder, which demonstrates that magnetic stimulation is capable of evoking NIRS signal changes not directly related to cerebral hemodynamic responses. The HbT waveforms measured on the stimulated shoulder were similar to the ones recorded on the stimulated hemisphere and to the waveforms of the circulatory parameters as characterized by correlation coefficients. In previous NIRS studies, decreases in HbT or HbO_2_ concentrations qualitatively similar to the ones presented here have been reported following TMS of the motor and prefrontal areas. The decreases have been measured above both the stimulated [3], [5] and contralateral [2], [4], [5], [7] cortices as well as anterior to the stimulation site [10]. The present study challenges the view that cerebral hemodynamic responses are the sole contributor to TMS-evoked NIRS signals. Based on the present results, magnetic-stimulation-evoked NIRS signals include physiological changes that are caused by TMS but do not result from the activation of cerebral neurons. Thus, the traditional few-channel NIRS measurement cannot be used to draw inferences about TMS-related brain activity without carefully designed control measurements and effective artifact removal methods.

**Figure 4:**
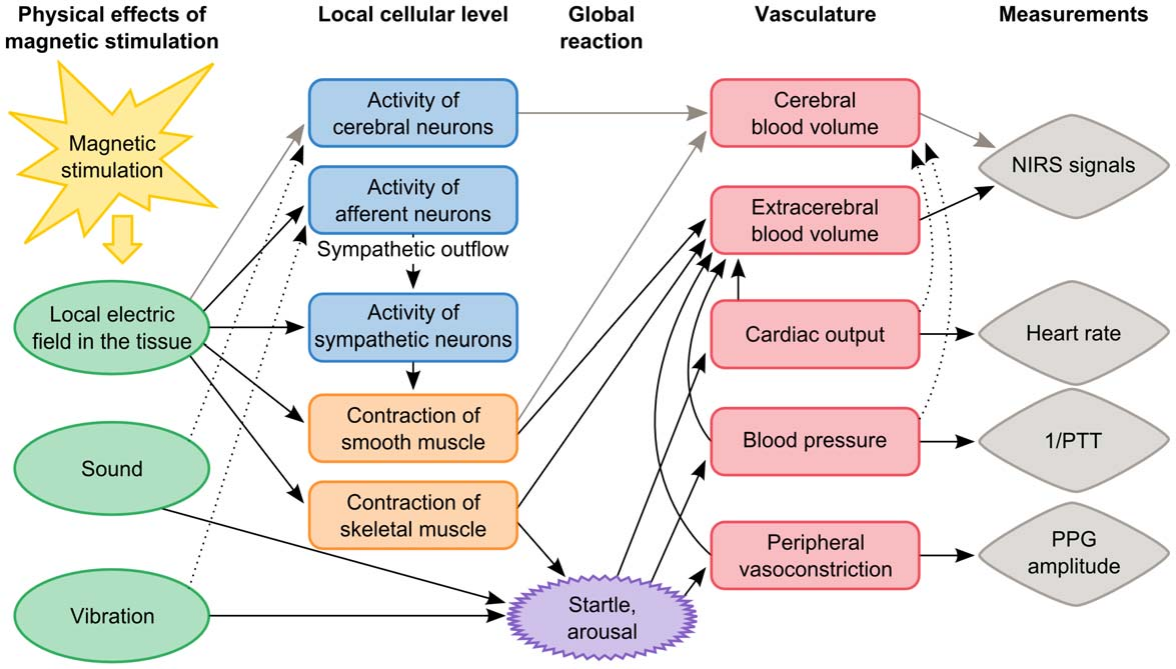
Effects of magnetic stimulation and their relation to measured parameters. Black solid lines indicate relations present in both brain and shoulder stimulation and gray solid lines relations present only in brain stimulation. Relations marked with dotted lines are not considered meaningful in terms of interpretation of the data.

Irrespective of the origin of the magnetic-stimulation-evoked HbT decrease on the stimulated shoulder, it is created by vasoconstriction. This is because HbT concentration is proportional to the blood volume in the measured tissue assuming a constant hematocrit [35]. There are at least four possible scenarios how magnetic stimulation can cause this vasoconstriction: 1) arousal and subsequent vasoconstriction in the skin, 2) direct stimulation of the smooth muscle walls of blood vessels and their contraction in the extracerebral or the cerebral tissue or both, 3) stimulation of sympathetic efferent or afferent nerve fibers, whose activation causes vasoconstriction either by directly activating the vascular smooth muscles or indirectly through sympathetic outflow, or 4) direct stimulation of skeletal muscles causing their contraction and, because of the pressure generated by this, constriction of blood vessels in the proximity of the muscles. Based on the present data, we consider explanations 2 and 3 the most probable ones, but all of them may sum up to produce the total NIRS response (Figure 4). The possible explanations are discussed in the following.

1) Arousal: A systemic arousal effect caused by the stimulation is evident in the systemic data: the PPG amplitude decreased in both brain and shoulder subjects, while 1/PTT, which is linked to blood pressure, increased in shoulder subjects. The changes in the PPG amplitude and the 1/PTT indicate that the vascular distensibility in the finger decreases and arterial stiffness in the upper extremity increases, both of which can be associated with vasoconstriction. The simultaneously decreased heart rate can be explained as a parasympathetic reflex to the slight elevation in blood pressure. Preliminary results of bilaterally measured circulatory parameters in one shoulder subject show comparable PPG amplitude and 1/PTT responses between the right and left hand, suggesting that the effect seen in the circulatory parameters is global. This kind of systemic circulatory changes have been reported to affect NIRS signals [22]–[24]. Indeed, the HbT waveforms in the brain and shoulder correlated with some of the circulatory parameters. However, it is unlikely that arousal alone produces the recorded HbT concentration changes in the shoulder experiment, since the responses on the stimulated and contralateral shoulders differ in polarity. If the HbT responses were solely caused by arousal, they should have the same characteristics in both shoulders because arousal acts globally. A systemic component may still be present in the signals measured on the stimulated shoulder if it is masked by a local component.

2, 3) Stimulation of smooth muscle walls of blood vessels or sympathetic nerve fibers: The difference in the polarity of the HbT responses between the stimulated and contralateral shoulders suggests that a local effect is included in the shoulder responses. The local effect may arise from direct stimulation of the smooth muscle walls in blood vessels, since the changing magnetic field induces currents in all conducting material, also in muscle fibers. The contraction time of vascular smooth muscles is in the order of seconds [36], which corresponds to the duration of the observed HbT responses. In addition to direct muscle activation, vascular smooth muscles may be activated indirectly via nerve fibers located near the target site. This could be brought about either by direct activation of efferent sympathetic vasoconstrictor nerve fibers or by reflex sympathetic outflow aroused by stimulation of nearby afferent nerve fibers, resulting in vasoconstriction at the target site and close to it. By this means, the stimulation could produce vasoconstriction that is local but covers a larger area than that just below the target site (e.g., the whole arm). The sympathetic nerve fiber activation could thus explain the changes seen in the PPG amplitude and the 1/PTT measured in the shoulder experiment. However, based on preliminary results on one shoulder subject who had comparable responses in a bilateral PPG measurement and the fact that also brain subjects showed a consistent decrease in PPG amplitude, it seems that the PPG amplitude and 1/PTT reflect arousal rather than stimulation-induced local sympathetic nerve fiber activity.

4) Skeletal muscle stimulation: Magnetic stimulation activates muscles near the stimulation coil [37]; therefore, it may produce a NIRS component that is related to skeletal muscle contraction. This component should, however, be small because TMS-evoked electroencephalography signals are often free of muscle artifacts following M1 stimulation at intensities greater than the ones in this study, even at 120% of the resting motor threshold [38]. The component stemming from muscle contraction should be small also because the stimulation site does not contain muscles and the subjects did not report any muscle contraction. In addition, as opposed to smooth muscles, the contraction of skeletal muscles lasts typically only tens or hundreds of milliseconds [36]; thus, the latter could not explain the slow HbT responses.

The brain and the shoulder differ in their anatomy and physiology, so direct conclusions about the origin of the NIRS responses following brain stimulation cannot be drawn based on those following shoulder stimulation. Nevertheless, only by stimulating some other area than the brain, the contribution of cerebral hemodynamic responses in the NIRS signals can be excluded. In addition, direct magnetic stimulation effects can only be studied by recording NIRS above the target site. The shoulder stimulation produced on the target site HbT responses, which correlated with those produced by the brain stimulation. This result is, despite the differences between the stimulation sites, a strong indicator of components not related to cerebral hemodynamic responses in the brain experiments. Any of the possible causes for the stimulation-related HbT concentration changes in the shoulder can produce physiological artifacts in the HbT responses in the brain experiments in a similar manner. Moreover, there is a discrepancy between TMS–NIRS and TMS–fMRI or TMS–PET studies, which can be explained by a component in the NIRS signals not related to cerebral hemodynamic responses; TMS–fMRI and TMS–PET have shown increases or no significant changes in cerebral blood flow [13], [15], [39] or blood oxygen level dependent (BOLD) responses [26], [40]–[42] on the stimulated hemisphere with subthreshold TMS intensities in contradiction with HbT decreases recorded in this and other NIRS studies [3], [5].

Differences in the HbR responses in the brain and shoulder may reveal some brain activity-related components. Summation of a cerebral hemodynamic response and a magnetic-stimulation-induced artifact in the NIRS signals recorded from the brain might explain the difference in the relative amplitude changes of HbO_2_ and HbR between the brain and the shoulder experiments, although differences in tissue structure and oxygenation can play a role as well: as the oxygen saturation of the response generating tissue decreases, the HbR concentration and the amplitude of the HbR response increases.

The contralateral HbT signals in the brain may also include components not directly related to cerebral neuronal activity. The recordings from the channels with the shortest source-to-detector distance suggest that the HbT responses include a decrease in the extracerebral layers because changes in the brain contribute minimally to this channel [30]. The decreased HbT concentration on the contralateral M1 is, however, consistent with the results of TMS–fMRI and TMS–PET studies, where negative BOLD responses [26], [41] and decreased regional cerebral blood flow [13] have been reported following subthreshold M1 stimulation. Since fMRI and PET have good spatial resolution, the reported hemodynamic changes are local, and extracerebral and cerebral signals are better separated than in NIRS, it is probable that the HbT decreases measured with the intermediate- and long-source-to-detector distance channels include an actual cerebral hemodynamic response, resulting from inhibited contralateral cerebral activity, rather than an effect of TMS on the vasculature unrelated to cerebral activity.

If we understand the nature of the different NIRS components, it may be possible to separate the cerebral-activity-induced hemodynamic response from the other components. Established methods for removing physiological artifacts from NIRS responses are particularly suitable for removing global signal changes. Principal component analysis (PCA), for example, divides the signal into uncorrelated components and the component with the largest eigenvalue reflects, in some cases, the systemic contribution [43], [44]. In the current study, this variation of PCA is not suitable because it seems that the stimulus-related components observed here are not completely of global origin but result also from local effects of the stimulation. In general, applying PCA-based artifact removal methods for TMS-evoked NIRS signals is problematic because the cerebral hemodynamic responses and other components are temporally and spatially correlated and thus cannot be easily separated with PCA. This temporal correlation between the hemodynamic response and the other components is also a problem in other correlation-based methods, such as the superficial signal regression in which the mean of the signals in channels with a short source-to-detector distance is decorrelated from the data with linear regression [45]–[47]. If the local effect of TMS is not only superficial but also prominent in deeper layers, superficial signal regression and other methods relying on signals reflecting activities in different layers in different proportions are not reliable. Indeed, if TMS causes direct contraction of blood vessels in the brain, it may be impossible to separate the resulting physiological artifact from the cerebral hemodynamic responses. Nevertheless, if this is not the case, sophisticated independent component analysis methods combined with a dense NIRS grid covering a large brain area and allowing reconstructions of the imaged volume to better spatially separate between different components could help in distinguishing the cerebral hemodynamic response [46], [48], [49]. In addition, it may be possible to draw inferences about TMS-evoked cerebral activity by carefully controlling the study design or by performing control measurements that would evaluate the effects of TMS-related physiological artifacts on the NIRS responses.

In conclusion, NIRS can be easily combined with TMS to measure stimulation-evoked hemodynamic changes. These changes, however, include components not directly related to cerebral activity. Such components can result from local effects of TMS on the vasculature and possibly from a global arousal effect. Based on the current measurements, these components cannot totally be separated from cerebral hemodynamic responses without effective artifact removal methods. Altogether, when recording TMS-evoked cerebral activity with NIRS, the study should be carefully controlled for physiological artifacts in order to draw reliable inferences about cerebral activity.

## Supporting Information

Figure S1 **Changes in HbO_2_ (red) and HbR (blue) following brain stimulation.** HbO_2_ and HbR responses from the stimulated (left) and the contralateral (right) brain hemispheres at short (uppermost row), intermediate (center row), and long (lowest row) source-to-detector distance channels. The standard errors of mean are shaded with the corresponding color. Vertical lines indicate times at which the TMS pulses were given. HbO_2_ decreased on both the stimulated and the contralateral hemisphere. * *p*<0.05 (*t*-tests for the response amplitudes compared to baseline, *p*-values controlled for FDR).
(TIF)

Figure S2 **Changes in HbO_2_ (red) and HbR (blue) following shoulder stimulation.** HbO_2_ and HbR responses from the stimulated (left) and the contralateral (right) shoulders at short (uppermost row), intermediate (center row), and long (lowest row) source-to-detector distance channels. The standard errors of mean are shaded with the corresponding color. Vertical lines indicate times at which the magnetic pulses were given. HbO_2_ and HbR decreased on the stimulated shoulder. * *p*<0.05 (*t*-tests for the response amplitudes compared to baseline, *p*-values controlled for FDR).
(TIF)
