# Supplementary figures and images for: Magnetic-Stimulation-Related Physiological Artifacts in Hemodynamic Near-Infrared Spectroscopy Signals
Source: PLoS One. 2011 Aug 26;6(8):e24002. doi: 10.1371/journal.pone.0024002 (PMC3162598; doi:10.1371/journal.pone.0024002)

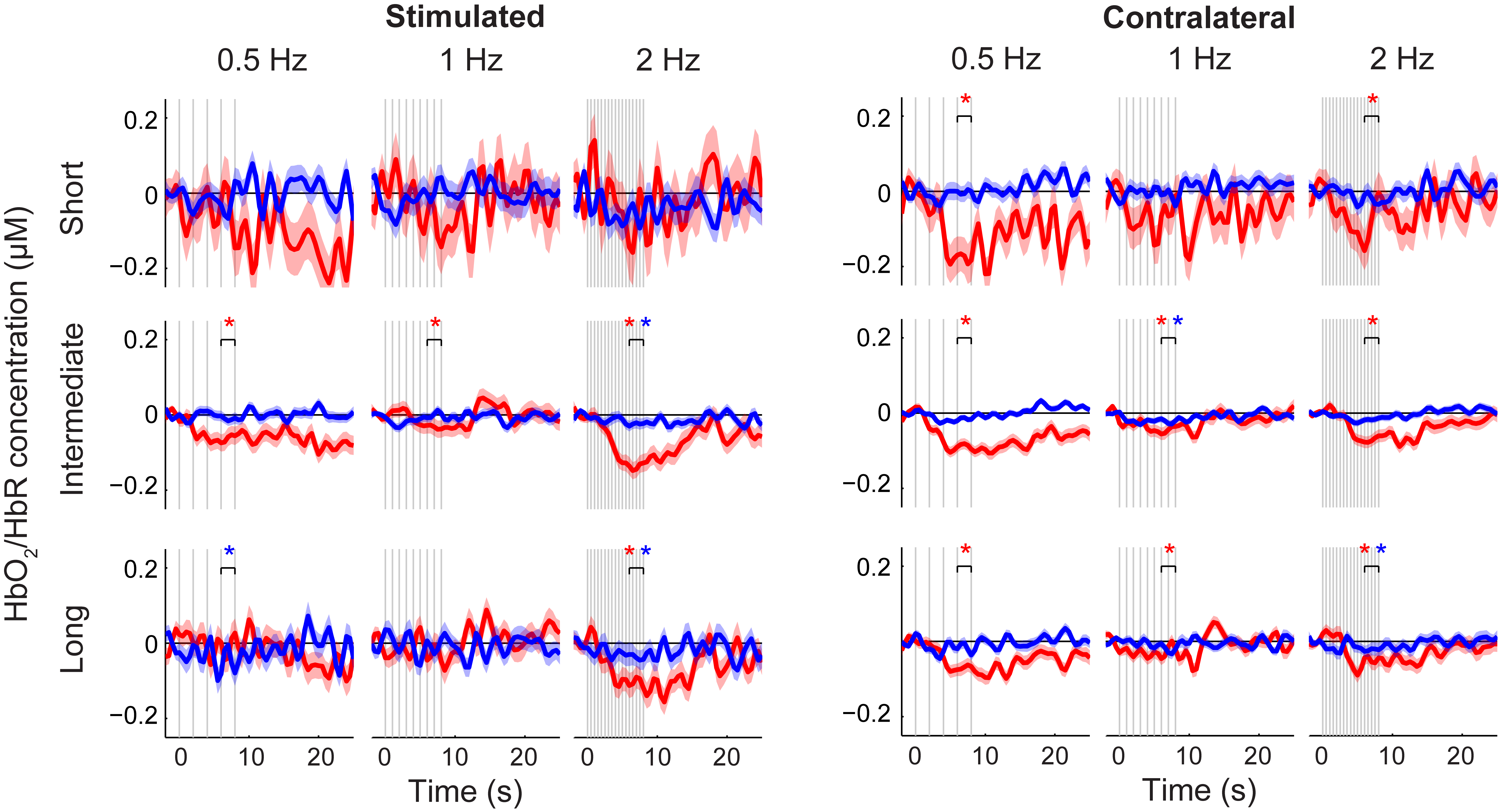

Supplement: Figure S1 — Changes in HbO2 (red) and HbR (blue) following brain stimulation. HbO2 and HbR responses from the stimulated (left) and the contralateral (right) brain hemispheres at short (uppermost row), intermediate (center row), and long (lowest row) source-to-detector distance channels. The standard errors of mean are shaded with the corresponding color. Vertical lines indicate times at which the TMS pulses were given. HbO2 decreased on both the stimulated and the contralateral hemisphere. * p<0.05 (t-tests for the response amplitudes compared to baseline, p-values controlled for FDR). (TIF) [file pone.0024002.s001.tif]

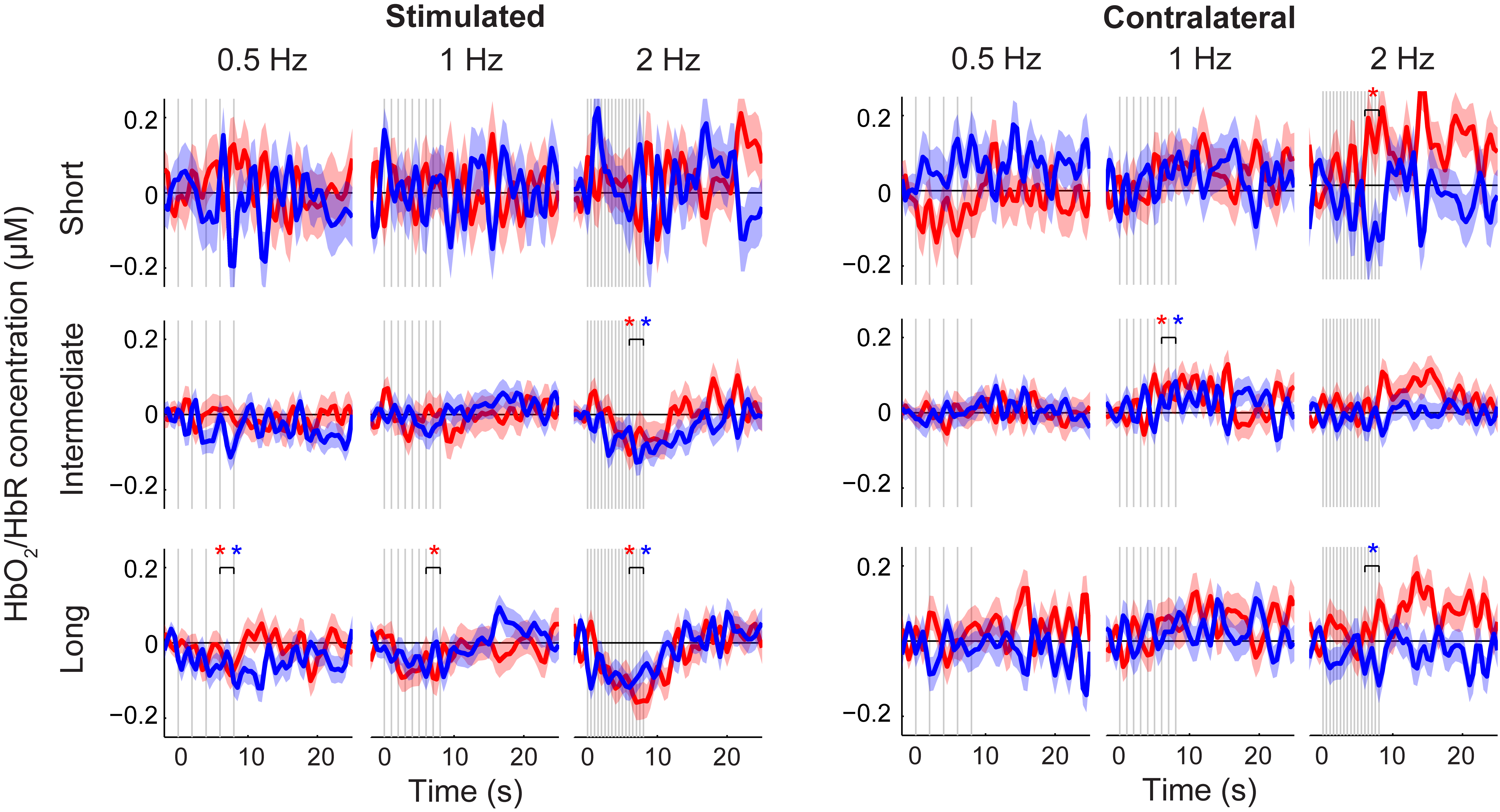

Supplement: Figure S2 — Changes in HbO2 (red) and HbR (blue) following shoulder stimulation. HbO2 and HbR responses from the stimulated (left) and the contralateral (right) shoulders at short (uppermost row), intermediate (center row), and long (lowest row) source-to-detector distance channels. The standard errors of mean are shaded with the corresponding color. Vertical lines indicate times at which the magnetic pulses were given. HbO2 and HbR decreased on the stimulated shoulder. * p<0.05 (t-tests for the response amplitudes compared to baseline, p-values controlled for FDR). (TIF) [file pone.0024002.s002.tif]
